# Evolution of a cluster of innate immune genes (β-defensins) along the ancestral lines of chicken and zebra finch

**DOI:** 10.1186/1745-7580-6-3

**Published:** 2010-04-01

**Authors:** Olof Hellgren, Robert Ekblom

**Affiliations:** 1 Edward Grey Institute, Department of Zoology, South Parks Road, Oxford, OX1 3PS, UK; 2 Department of Animal Ecology, Lund University, SE-22362, Lund, Sweden; 3 Department of Animal and Plant Sciences, University of Sheffield, Western Bank, Sheffield S10 2TN, UK; 4 Department of Population Biology and Conservation Biology, Uppsala University, SE-75226 Uppsala, Sweden

## Abstract

**Background:**

Avian β-defensins (AvBDs) represent a group of innate immune genes with broad antimicrobial activity. Within the chicken genome, previous work identified 14 AvBDs in a cluster on chromosome three. The release of a second bird genome, the zebra finch, allows us to study the comparative evolutionary history of these gene clusters between from two species that shared a common ancestor about 100 million years ago.

**Results:**

A phylogenetic analysis of the β-defensin gene clusters in the chicken and the zebra finch identified several cases of gene duplication and gene loss along their ancestral lines. In the zebra finch genome a cluster of 22 AvBD genes were identified, all located within 125 Kbp on chromosome three. Ten of the 22 genes were found to be highly conserved with orthologous genes in the chicken genome. The remaining 12 genes were all located within a cluster of 58 Kbp and are suggested to be a result of recent gene duplication events that occurred after the galliformes- passeriformes split (G-P split). Within the chicken genome, AvBD6 was found to be a duplication of AvBD7, whereas the gene AvDB14 seems to have been lost along the ancestral line of the zebra finch. The duplicated β-defensin genes have had a significantly higher accumulation of non-synonymous over synonymous substitutions compared to the genes that have not undergone duplication since the G-P split. The expression patterns of avian β-defensin genes seem to be well conserved between chicken and zebra finch.

**Conclusion:**

The genomic comparisons of the β-defensins gene clusters of the chicken and zebra finch illuminate the evolutionary history of this gene complex. Along their ancestral lines, several gene duplication events have occurred in the passerine line after the galliformes-passeriformes split giving rise to 12 novel genes compared to a single duplication event in the galliformes line. After the duplication events, the duplicated genes have been subject to a relaxed selection pressure compared to the non-duplicated genes, thus supporting models of evolution by gene duplication.

## Background

Gene duplication has been an important factor for shaping the immune defence against the high diversity of pathogens faced by vertebrates [1-4]. This can be seen in the gene clusters of the major histocompatibility complex (MHC) class I and II [2], but also in the innate immune system in, for example, genes coding for toll-[1] or anti microbial peptides, i.e. defensins [5].

Defensins are a group of small cationic peptides that play an important role in the innate immune system of vertebrates, as well as in invertebrates [6-8]. Defensins provide a broad pathogenic defence against bacteria, viruses, fungi as well as showing activity against protozoan parasites [9-11], primarily by binding to and disrupting the membrane of the invading organism. However, recent studies have shown that they also might play an important role in the link between other parts of the immune system by signalling to macrophages, lymphocytes and mast cells [12,13]. Moreover, the peptides might play a more complex role in destroying pathogens by not only disrupting cell membranes, but also by being able to block viral infections by disrupting the replication cycle or altering host cell recognition sites [10,14]. Depending on the distribution of sulphide bonds within the mature peptide, defensins can be divided into three subfamilies; θ-, α- and β-defensin. In the chicken genome, 14 genes coding for β-defensins have been found in a dense cluster located on the end of the 3^rd ^chromosome (3q3.5-q3.7, [15-17]). These genes consist of four exons (E1-E4), E1 coding for 5'UTR, E2 the signal peptide and a part of the propiece (i.e. the part of the prepeptide that is later cleaved off to get the mature peptide), E3 codes for the rest of the propiece and the mature peptide and the fourth exon codes for the 3'-UTR. β-defensins are expressed and excreted by neutrophils and epithelia cells lining various organs [18-20]. In chicken, the tissue-specific pattern seems to vary across the different defensin genes with some showing expression in a wide array of tissues (e.g. AvBD9 with found expression in 22 different tissues, ranging from brain to different intestines and testis [16]), whereas other seem to have more limited expression patterns (e.g. AvBD8 with expression only observed in the liver and gall-bladder [16]). For complete summary, see [16]. Tissue specific patterns might however be hard to fully understand, without experimental exposure to pathogens, as the genes as a group, seems to vary in terms of having constitutive or induced expression patterns [21-24]. Constitutively expressed AvBDs can potentially be observed in all tissues in which they are active in, whereas AvBDs with induced expression would only be observed when exposed by pathogens or other external triggers, thus possibly biasing comparisons of tissue specificity between different AvBDs.

Evolutionary studies have shown that the formation of defensin genes has been driven by ancient gene duplication events where, for example, all α-defensins are thought to have evolved by gene duplication after the bird-mammal split and the θ-defensins to have arisen in the primate lineage, originating from the α-defensins [17]. A comparison of β-defensin genes across distant species (i.e. birds and mammals) yields weak phylogenetic signals of species [17], suggesting that many of these genes have been duplicated and became defined in function long before the bird-mammal split. However, a comparison of more closely related mammal species has yielded evidence of more recent gene duplications [25].

By comparing the chicken genome with a more closely related species, the zebra finch, we investigated how β-defensin genes have been evolving since the G-P split in the late Cretaceous period, i.e. about 100 million years ago [26], in terms of duplication events, gene losses and rates of evolution. More specifically, we tested whether the gene cluster of β-defensins have been conserved across the ancestral lineages of the birds species, and the extent to which the avian β-defensin complex had acquired novel genes through duplications along the two ancestral lineages. We also tested whether duplicated zebra finch β-defensin genes where evolving according to different selection pressures compared to unduplicated genes and investigated tissue specific expression patterns of this gene cluster using a digital transcriptomic approach [27,28]

## Results

Throughout the paper the nomenclature for avian β-defensins (AvBDs) proposed by Lynn *et al*. (2007) is used, where avian β-defensins (also known as gallinacins) found in the chicken formally named Gall1-14 have been renamed to AvBD1-14 [15]. Genes found in the zebra finch that have ortholog chicken defensins are numbered accordingly but with z as a suffix in order to tell them apart and newly found genes without an orthologous chicken gene named with a number greater than 100.

**Figure 1:**
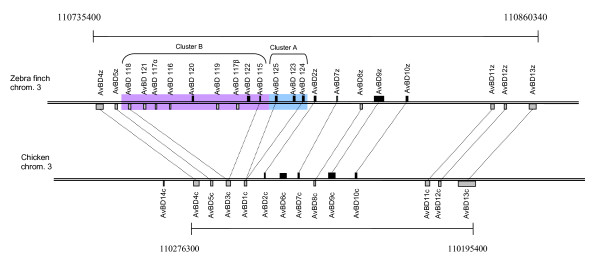
**Location of β-defensin genes on chicken chromosome 3 and zebra finch chromosome 3**. The scales are the same for both chromosomes. The genes are drawn above or below depending on whether they are located on the plus or minus strand on the chromosome. The size of the genes is based on the start of exon 2 to the end of exon 3 (i.e. the start of the signal peptide to the end of the mature peptide).

**Figure 2:**
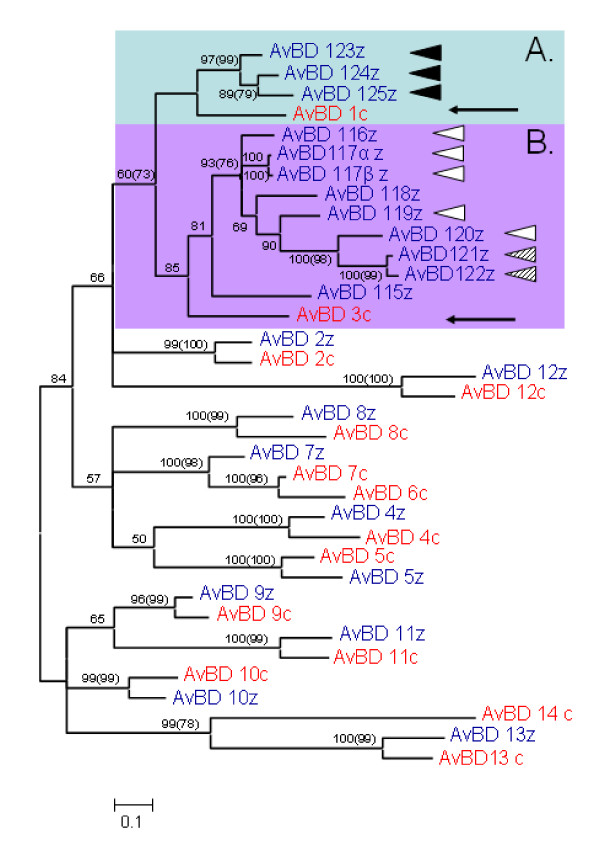
**Bayesian major consensus tree of the relationship between AvBD genes found in the chicken and zebra finch genome**. Genes in blue ending with z denote lineages found in the zebra finch genome and genes marked in red and a c-suffix denotes genes found in the chicken genome. Posterior probabilities are displayed at the branches and corresponding bootstrap values above 50 obtained from neighbour-joining method is displayed in brackets). Highlighted Cluster A and B refer to two clusters of duplicated genes, presumably originating from the genes AvBD1 and 3 (see arrows). Genes with identical signal peptides are noted with similar triangles to the right of the sequence name. The tree is calculated as unrooted and then rooted on mid-point.

**Figure 3:**
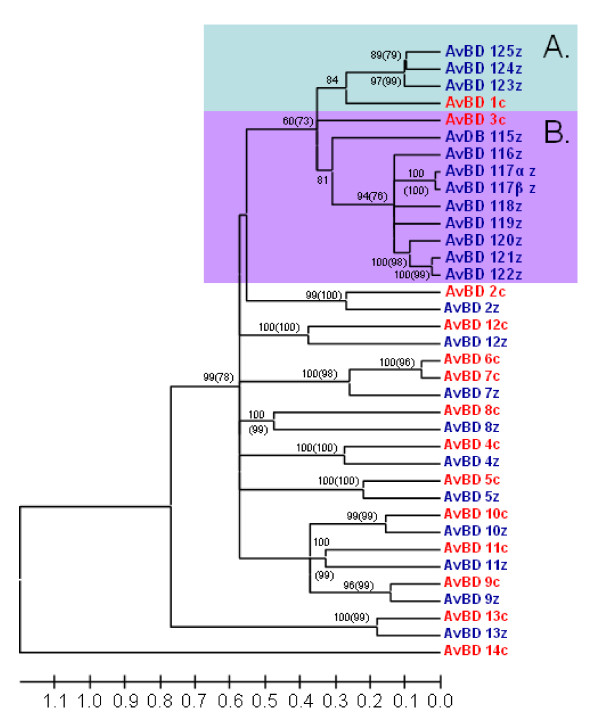
**Linearized tree with topology and posterior probabilities/bootstrap values as the tree in Figure 2 but branch lengths are based synonymous sites only**. Genes in blue ending with z denote lineages found in the zebra finch genome and genes marked in red and a c-suffix denotes genes found in the chicken genome. Posterior probabilities are displayed at the branches and corresponding bootstrap values above 50 obtained from neighbour-joining method is displayed in brackets). Highlighted Cluster A and B refer to two clusters of duplicated genes, presumably originating from the genes AvBD1 and 3. The tree is calculated as unrooted and then rooted on mid-point. The tree is calculated as unrooted and then rooted on mid-point.

**Figure 4:**
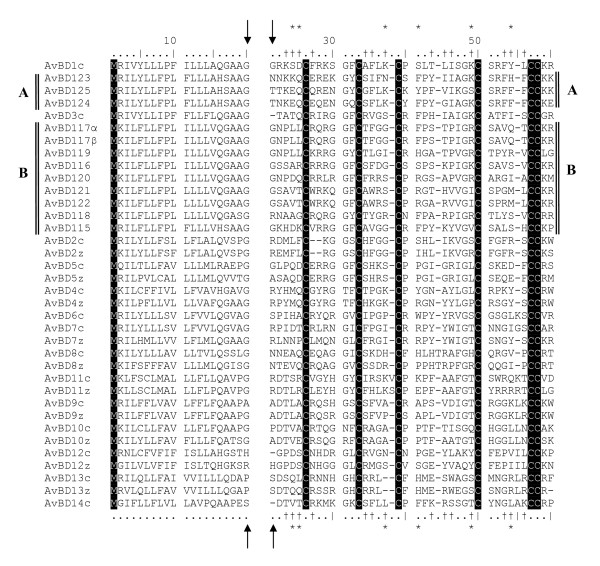
**Alignment of the amino acid sequences of chicken and zebra finch β-defensins**. Arrows indicate end of the signal peptide and the start of the mature peptide. Conserved sites are highlighted in black. Sites indicated by "*" have been found to be under positive selection in the chicken genome [15] and sites with "†" show evidence of positive selection in cluster B in the zebra finch genome (see also figure 2).

The zebra finch genome contains at least 22 different genes coding for β-defensins. All 22 genes cluster within 125 Kbp on the 3^rd ^chromosome (figure 1, table 1). Ten of these have an ortholog gene in the chicken genome. In these cases the zebra finch genes were grouped together, with a "sister gene" from the chicken genome, with a posterior probability > 95% (Baysian phylogeny) or a bootstrap value > 95 (neighbour-joining tree) (figure 2 and 3). Further support of the relationship between the genes can be found when investigating the gene location and order of the two sets of genes (figure 1) where the 10 orthologous genes have kept their orientation and relative location in comparison to each other on the chromosome. This suggests that these genes have been duplicated long before the G-P split and have thereafter been conserved along the ancestral lineages of the two different bird species. In the case of AvBD6 found in chicken, it seems to have originated by a duplication of AvBD7 after the G-P split (figure 2 and 3), whereas the AvBD14 seem to have been lost in the ancestral line of the zebra finch. The remaining 12 genes found in the zebra finch genome stem from two ancestral genes, likely AvBD1 and AvBD3 based on the phylogenetic relationship, which correspond to the physical location of the genes on the chromosome (figure 1, 2 and 3). The 12 unique zebra finch genes were all found within a cluster located between gene AvBD2 and AvBD5, the same location where AvBD1 and AvBD3 are located in the chicken genome. The gene cluster can be divided into two separate groups, one consisting of AvBD123-AvBD125 (referred to as cluster A) that clustered together with AvBD1, and where all three genes shared identical signal peptide (figure 2, 3 and 4). The second cluster (cluster B) consists of 9 genes located next to each other (AvBD115-122) on the chromosome (figure 1) and clustered together in the phylogeny (figure 2, 3 and 4); these probably share an ancestral origin with AvBD3. Five of the genes in cluster B share one type of signal peptide, whereas two others (AvBD121 and 122) share a signal peptide that differs by one amino acid substitution (figure 2, 3 and 4). All other β-defensin genes found in the zebra finch genome had unique signal peptides. Within cluster B two sets of genes are likely to be the product of very recent gene duplication events. The genes AvDB117α and β share identical amino acid composition for signal and mature peptide and differ only at the nucleotide level (including the signal and mature peptide sequences), with one synonymous substitution in the signal peptide and an additional 21 SNP when including the intron, resulting in a 3.6% difference along the full gene. The other case is AvBD121 and 122 that differs at three different amino acid positions in the mature peptide (figure 4) and has three synonymous substitutions, two of which are in the signal peptide. We can't exclude that the case of AvBD117α and β is a misassemble of two different alleles or a case of gene copy variation, as seen in human β-defensins [29]. However, that they do not map next to each other (figure 1) together with the fairly diverged intron reduces that chance. It would therefore be of importance for future study to investigate the occurrence of gene-copy variation, not only for AvBD117 but also for the gene group as a whole.

**Table 1:** Avian β-defensins found in both the chicken and zebra finch genomes with their corresponding GenBank numbers.

A-defensin	Length chicken	Length zebra finch	GeneBank nr chicken	GeneBank nr zebra finch	Net charge ph 7.0	Isoelectric point	Average hydrophility
AvBD1	700		NM_204993		7.7	9.9	0.1

***AvBD 123***		544		BK006977	5.8	9.2	0.1

***AvBD124***		543		BK006978	0.7	7.6	0

***AvBD125***		545		BK006979	5.7	9.2	0.1

AvBD2c	300	459	NM_204992		3.9	8.6	-0.3

AvBD2z		459		BK006967	4.9	8.9	-0.1

AvBD3	1200		NM_204650		5.8	9.4	-0.2

***AvBD115***		254		BK006966	5.9	9.2	0

***AvBD116***		449		BK006965	6.7	9.9	0.3

***AvBD117α***		611		BK006961	5.7	9.4	-0.1

***AvBD117β***		611		BK006962	5.7	9.4	-0.1

***AvBD118***		603		BK006963	7.7	10	0.1

***AvBD119***		641		BK006964	5.8	9.3	-0.2

***AvBD120***		689		BK006981	7.7	11	0.3

***AvBD121***		706		BK006975	5.8	9.4	-0.2

***AvBD122***		710		BK006976	7.7	10.6	0

AvBD4c	1350		NM_001001610		5.8	9.1	-0.3

AvBD4z		1944		BK006982	6.8	9.4	-0.1

AvBD5c	600		NM_001001608		1.8	8	0.3

AvBD5z		783		BK006969	0.8	7.6	0.3

AvBD6c	1650	-	NM_001001193		5.8	9.2	-0.6

AvBD7c	400		NM_001001194		4.7	8.9	-0.2

AvBD7z		268		BK006970	5.7	9.2	-0.3

AvBD8c	500		NM_001001781		1.1	7.7	0.1

AvBD8z		700		BK006968	2.8	8.3	0.2

AvBD9c	1800		NM_001001611		3.8	8.6	0

AvBD9z		2544		BK006972	3.7	8.6	0

AvBD10c	500		NM_001001609		1.8	8	-0.2

AvBD10z		440		BK006971	1.8	8	0

AvBD11c	1200		NM_001001779		4.8	8.9	0

AvBD11z		1095		BK006973	3.9	8.6	0

AvBD12c	700		NM_001001607		-0.2	6.8	0

AvBD12z		731		BK006980	-1.1	6	-0.3

AvBD13c	4700		NM_001001780		4	8.6	0

AvBD13z		1919		BK006974	4.9	8.9	0.5

AvBD14		-	AM 402954		6.7	9.6	0.1

Lengths correspond to the start of the signal peptide to the end of the mature peptide.

Nine out of the 22 zebra finch β-defensin loci showed evidence of expression in at least one out of six sampled tissues (Table 2). A total number of 2,338 sequence reads were mapped to one of these genes. Levels of gene expression varied dramatically between the different loci with high expression levels in AvBD9 and AvBD10 and low expression of the other seven. All genes had high level of tissue specificity of gene expression (τ). The expression profile also varied between loci, with AvBD8, AvBD9 and AvBD10 being expressed mainly in liver and weakly in embryo, AvBD123 was expressed exclusively in testes, while the rest of the loci were expressed mainly in skin and/or spleen. None of the genes had any detected expression in muscle.

**Table 2:** Expression of nine zebra finch β-defensin loci in six different tissues sampled.

Locus	Embryo	Liver	Muscle	Skin	Spleen	Testes	τ
AvBD2	0.0	7.6	0.0	83.2	59.0	0.0	0.630
AvBD7	0.0	0.0	0.0	0.0	13.9	0.0	0.737
AvBD8	3.1	7.6	0.0	0.0	0.0	0.0	0.616
AvBD9	6.2	4357.5	0.0	4.0	41.7	23.4	0.743
AvBD10	43.2	1338.8	0.0	4.0	3.5	20.0	0.720
AvBD13	0.0	0.0	0.0	7.9	0.0	0.0	-
AvBD115	0.0	0.0	0.0	0.0	6.9	0.0	-
AvBD123	0.0	0.0	0.0	0.0	0.0	3.3	-
AvBD125	0.0	0.0	0.0	0.0	6.9	0.0	-

total library size:	323897	392890	325646	252349	287902	299755	

Values given represent numbers of transcripts (454-sequencing reads) per million (TPM). Tissue specificity of expression (τ) for genes represented by at least four transcripts was calculated according to Mank et al. (2008) [41].

**Figure 5:**
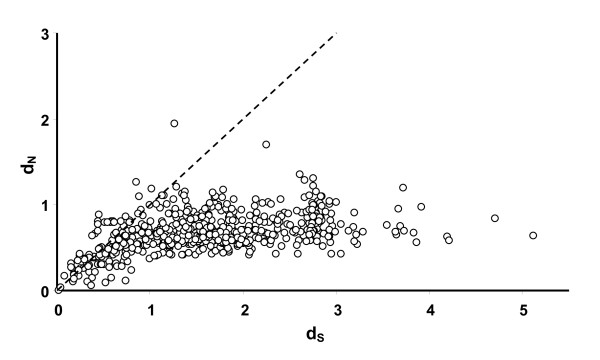
**Relationship between pairwise d_N _(rate of non-synonymous substitutions) and d_S _(synonymous substitutions) between all sequences in figure 3**. The line represent the ω = 1 i.e. neutral selection.

The d_N_/d_S _ratio (ω) calculated pairwise over all obtained genes together with chicken defensins showed evidence of purifying selection, i.e. values of ω < 1 (mean dS = 1.59 S.E. = 0.03, mean dN = 0.54, S.E. = 0.01, mean ω = 0.67 S.E. = 0.01, figure 5). However, looking at the distribution of the ω values in Figure 5 together with having a very deep phylogenetic tree (figure 2) indicate that the faster evolving non-synonymous sites have become saturated, thus making comparisons between paralog genes in the tree biased. For the following analysis we therefore only used pairwise comparisons between ortholog genes (i.e. for the same gene but between species), or comparisons between recently duplicated genes after the G-P split.

**Figure 6:**
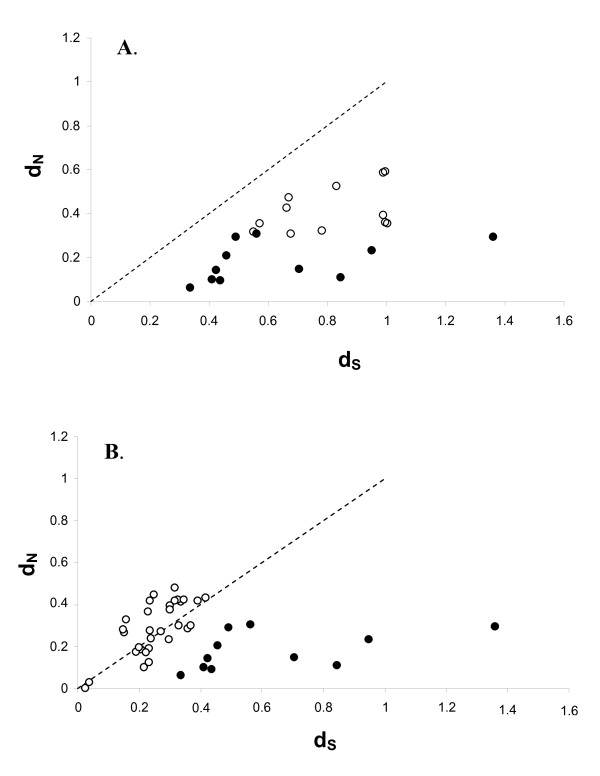
**Relationship between pairwise d_N _(rate of non-synonomous substitutions) and d_S _(synonymous substitutions)**. The line represent ω = 1 i.e. neutral selection. *A*) White dots represent pairwise values obtained between AvBD1 and AvBD3 with their respective cluster of orthologous genes (see cluster A and B in fig 2). Black dots represent pairwise values between the non-duplicated orthologous genes (i.e. comparison between the chicken and zebra finch genes outside cluster A and B). *B) *White dots represent pairwise values obtained between duplicated genes in phylogenetic well supported clades. Black dots represent pairwise values between the non-duplicated orthologous genes (i.e. comparison between the chicken and zebra finch genes outside cluster A and B).

Pairwise comparisons between AvBD1/AvBD3 and its duplicated homolog genes in the zebra finch genome showed higher values of ω than comparisons between orthologous chicken - zebra finch genes that had not undergone duplication (mean ω = 0.53 (S.E. = 0.04) vs. mean ω = 0.3 (S.E. = 0.05), Mann-Whitney U-test, Z = -3.26, P < 0.001, Monte Carlo, test P < 0.001, Figure 6a). Testing the rate between more recent and phylogenetically more robust clusters of duplicated genes in the zebra finch genome in relation to the rate of evolution of the unduplicated orthologous genes revealed an even bigger relaxation of the selection pressure of the duplicated genes (mean ω = 1.0 (S.E. = 0.08) vs. mean ω = 0.3 (S.E. = 0.05), Mann-Whitney U-test, Z = -4.6, P < 0.0001, Monte Carlo, test P < 0.001, Figure 6b). The mean value of ω for the duplicated genes was close to unity, indicating neutrality, although several cases are observed with ω > 1 (Figure 6b). One reason for neutral evolution to occur may be if the genes have lost their function after the duplication events and thus any selective forces acting upon it. However, if only a few amino acid positions are responsible for the differences in antimicrobial properties of the peptide, positive selection acting upon these specific sites might be might be masked by negative selection acting on other sites that keep the structure of the peptide.

Relaxed selection pressure might also have resulted from those genes in the gene family having become pseudo-genes causing them to evolve under neutrality. However, in no case were premature stop codons or frame shifts observed in the investigated exons. Another way to investigate whether the genes are still active would be to test if genes originating from one of the ancient genes still show evidence of positive selection on specific amino acid sites and, if so, whether these sites are corresponding to those of the "old active genes." Testing for specific sites under positive selection (likelihood ratio test, LRT) showed that the best model of selection for the cluster B data was model M8, i.e. the model that allowed for certain amino acid positions to be under positive selection (Table 3 and 4). Six amino acids were found to have evolved under positive selection in the zebra finch β-defensins (figure 4). The methods of codon-based selection have been strongly criticised lately [30,31]. However, in the case of β-defensins in the chicken genome the changes in amino acid positions found to be under positive selection have proven to generate novel pathogen specific responses [32], thus implying that the codons picked by the model do represent sites under "true adaptive selection." All amino acids identified in our model (with one exception) have previously identified as being under positive selection in chicken [15]. All of the selected amino acids were located within the mature peptide. The probability of the model to pick the 5 out of the 14 (the number of positively selected sites in chicken) selected amino acids over all variable sites in the signal and mature peptide is lower than 0.01.

**Table 3:** Likelihood ratio test between different models of evolution acting on genes in cluster B of duplicated zebra finch β-defensin genes (fig 2).

LRT test	d.f.	2Δl	P (χ^2^)
M1a vs. M2a	2	24.28	< 0.001
M8a vs. M8	1	18,46	< 0.001*
M7 vs. M8	2	20.42	< 0.001

* calculated as P = χ^2^_1 _(2Δl)/2: see Yang 2007 [58]and Swanson *et al*. (2003) [61].

**Table 4:** Parameter estimates and Log-likelihood values, under models of variable ω among sites, and selected sites under positive selection in cluster B of duplicated zebra finch β-defensin genes.

Model	np	Parameters	ML	d_N_/d_S_	Amino acid positions under positive selection (BEB, > 0,95*. > 0.99**)
M1a (nearly neutral, ω = 1 or < 1)	2	p_0 _= 0.39, ω_0 _= 0 p_1 _= 0.61, ω_1 _= 1	-966.38	0.61	

M2a (selection, allows ω > 1)	4	p_0 _= 0.38, ω_0 _= 0 p_1 _= 0.27, ω_1 _= 1 p_2 _= 0.35, ω_2 _= 3.67	-954.24	1.56	24**, 25**, 35*, 37*, 43*, 45**, 53*, 56**

M8a (β dist and ω = 1)	4	p_0 _= 0.39 p = 0.005 q = 99.00 p_1 _= 0.61, w = 1.00	-966.38	0.61	

M7 (β dist and ω 0-1)	2	p = 0.012, q = 0.005	-967.36	0.7	

M8 (β dist and ω > < 1)	4	p_0 _= 0.64, p = 0.005, q = 0.008, p_1 _= 0.36, ω = 3.65	-957.15	1.57	23*, 24**, 25**, 27*, 34*, 35**, 37**, 43**, 45**, 46*, 49*, 53**, 54*, 56**

## Discussion

Several of the genes included in the β-defensin cluster of the chicken have been highly conserved both in the zebra finch and in the chicken since they split around 100 million years ago. However, new avian β-defensin genes have been acquired through duplications along the two ancestral lineages, especially in the zebra finch genome. After the duplications, the new genes have evolved under relaxed purifying selection (although some amino acids show evidence of positive selection) compared to the non-duplicated genes.

The zebra finch genome contains a cluster of 22 β-defensin genes, of which ten were found to have orthologous genes in the chicken genome, originating from ancient gene duplications before the G-P split. The remaining 12 genes have evolved by a series of more recent gene duplication events. The long branches and low basal resolution in the phylogenies (figure 2 and 3) suggests that the different orthologous genes have occurred by ancient gene duplication events and then been conserved along the evolutionary lineages of the chicken and the zebra finch, i.e., in the region of 100 million years. That β-defensin genes arose by ancient gene duplications has been suggested by earlier studies [5,17,25]. The functions of the avian β-defensins in chicken are increasingly well understood, both in terms of where in the body they are being expressed but also how effective they are against different pathogens (for summary see [16,33]). The overall patterns of tissue specific expression of zebra finch AvBDs do coincide to large extent to that found in chicken (for detailed comparison see van Dijk 2008 [16] and table 2). Similar to chicken, AvBD2, 8, 9, and 10 was expressed in the liver, AvBD9 and 10 in testis and skin, AvBD2 and9 in spleen. The discrepancies are AvBD2 and 13 that in the zebra finch that also was found to be expressed in the skin, AvBD7 and 10 was additionally found in the spleen (table 2).

Within a small region of 56 Kbp in the zebra finch genome a series of more recent gene duplications have occurred. Within this short physical distance on chromosome 3, there are two AvDB genes in the chicken genome. These two genes have undergone several duplication events and can now comprise 12 different AvBD genes in the zebra finch genome. That these duplicated genes have not lost their function is indicated by several factors.

Firstly, our data on expressed genes together with a similar study on expressed genes in the brain of zebra finch [26] identify four or possible five of the duplicated genes (with two and three expressed genes from each of the duplication clusters). In this study, we identified the genes AvBD123 (expressed in spleen) and 125 (expressed in the testis) that are a result of a duplication event (Table 2). The third expressed gene belonging to duplication cluster identified in this study was AvBD115 (also with expression in the spleen). AvBD115 was together with AvBD117α/β also found to be expressed in the separate study of [26]. In their study the expressed sequence tag, FE7232851, was identical with AvBD115 and ESTs, DV954612.1 and FE728335.1, are likely alleles of AvBD 117α/β as it only differed by three SNPs, two of which have caused nonsynonomous substitutions at position 34 and 56 (figure 4) changing Threonine (T) to Serine (S, both amino acids with uncharged polar R groups) and FE728335.1 had an additional SNP causing a nonsynonomous substitution on position 41 changing an Phenylalanine (F) to Tyrosine (Y). It should be noted that a blast search of the EST against the zebra finch genome yielded AvDB 117α and β as the closest genes. A possible reason that some of the identified genes were not found to be expressed in the investigated zebra finches could be due to tissue specific expression in organs not investigated by us. In fact, in chickens several of the β-defensins are expressed in tissues not evaluated in our study. These are manly tissues associated with internal organs such as bursa, the intestines, lungs but also bone marrow and leucocytes [16]. Further, if no infection or immune response associated with the investigated genes have been initiated then we would not expect to see the gene being expressed.

Secondly, several of the genes share identical signal peptides, although the mature peptides have evolved new amino acid sequences (figure 2, 3 and 4). Thirdly, within cluster B the amino acids under positive selection (Table 3) are similar to the sites known to be under selection in the functional chicken AvBD genes (Table 3 and figure 2 and 3, [34]).

Several models have been put forward about how new genes and gene functions might arise through gene duplication. Initial models (different versions of the neo-functionalization model) [35,36] suggested that after duplication a gene is free from selection pressure and can thereby accumulate mutations that could lead to new functions. However, the main problem with these models is that they do not explain why the original (neutral) duplication is not lost by drift in the population [37,38]. If the original gene have evolved to conduct more than one function then the two functions can be divided between the two new genes thus escaping adaptive conflicts between the two functions (sub-functionalization models) [35,39,40]. Even here, the duplicated genes are released from selection until one of the functions has been lost, although this time might be shorter than in the neo-functionalization model, as in this case mutations have to cause losses of a function, instead of gaining new specialised ones [37]. However, a model (the innovation, amplification, duplication, IAD model), that maintains duplicated genes under continuous selection has recently been proposed by Bergthorsson and co-workers [37]. First, innovation, in which the original gene product has both a primary function and a secondary function which have neither deleterious nor beneficial properties. With a change in ecological niche, or for immune genes, the encounter of a new pathogen fauna, the secondary function becomes beneficial and an increase of the activity is selected for. In the second step, amplification, an increase of the secondary activity is achieved by gene duplication and thus selected for. Lastly, divergence, being freed from its original function the duplicated gene can now evolve improvements in what previously was a secondary function, thereby diverging from its original gene. In the present dataset there are several indications that the IAD model might be able to explain how the AvBD genes have duplicated and diverged in the zebra finch genome, as discussed below.

Individual AvBD genes have been shown to have activity against several different groups of pathogens [16,41]. The effectiveness against the different groups of pathogens might, however, differ between the different AvBD genes [41-43] thus depending on the environment having primary and secondary function against different pathogens. In two cases in the zebra finch genome very recent events of gene duplication can be observed, in one case the mature peptides are identical (i.e. AvBD117 α and β) and in the second case (AvBD121 and 122) the mature peptide is highly similar. If having multiple copies of an AvBD gene increases the gene expression, as found when investigating gene-copy variation of human β-defensins [29]. Then the effectiveness against certain pathogens might increases simply due to an increase in dosage, as seen in vitro [41]. Of interest would be to investigate whether the expression of the AvBD117 α and β is associated with an increased expression, in relation to other single copy genes. Once duplicated, the genes should start to diverge in order to be optimized for the function that was formerly only a secondary function. In the cluster of duplicated genes in the zebra finch genome selection has been relaxed (Figure 6a and 6b), compared to the genes that have not been duplicated. It should also be mentioned that in the case of the more recent duplication event in the chicken genome (AvBD6 and 7, figure 2) strong positive selection has acted upon the genes since the duplication event (pairwise comparison, ML, ω = 1.8). As defensins have been found to have broader functions such as signalling to the innate immune system [12,13,44], it might also be that the diversification of the duplicated genes in the zebra finch genome is associated with selection of other functions than direct disruption of pathogen cell membranes. In the chicken genome the expression patterns of the duplicated genes AvBD6 and 7 show similar tissue expression pattern [16], suggesting that they have retained similar functions and both have kept strong antimicrobial activity *in vitro *[16,43].

It can not be excluded that our finding that AvBD14 has been lost somewhere along the zebra finch lineage is an artefact from a misassemble of the zebra finch genome. It may also be that our method for finding β-defensin genes was not general enough to find this gene. Furthermore, AvBD14 is yet to be annotated in the chicken genome (although the coding sequence for this gene was deposited in GenBank in 2006 there is still no publication describing the finding of this gene). However, an ortholog gene to AvBD 14 has been identified in the Turkey genome indicating that the chicken AvBD14 is not an assembly artefact (David Burt, pers. comm.)

We have shown here, together with previous studies on mammals and frogs, that antimicrobial peptides such as *β*-defensins are evolving through gene duplication and positive selection in vertebrates [45,46]. This stand in contrast the counterpart of AMPs in the immune system in insects (i.e. *Drosophila *spp.) where studies several times failed to detect positive selection, although duplications seems to have been common [47-49]. As insects lack the adaptive immune system, selection might work on other mechanisms that regulate the gene expression instead of making the peptides more specific [49]. To fully understand the different evolutionary pressures imposed on the immune system of different organisms, we call for studies that examine not only the selective forces acting upon the peptide itself but also studies examining the selection on regulatory elements associated with the peptide expression.

Studying the evolution of immune genes is associated with additional challenges compared to other genes as they might lose functionality by not evolving as pathogens constantly are evolving in order to evade the function of the immune genes. However, as shown in this study, the steady increase in the availability of genomic data makes comparative studies possible in order to understand the underlying mechanisms of evolution and diversification of genes in the immune system.

## Conclusion

The genomic comparisons of the β-defensins gene clusters of the chicken and zebra finch illuminate the evolutionary history of this gene complex. Along their ancestral lines several gene duplication events have occurred in the passerine line after the galliformes-passeriformes split giving rise to 12 novel genes compared to a single duplication event in the galliformes line. After the duplication events the duplicated genes been subject to a relaxed selection pressure, compared to the non-duplicated genes.

## Method

### Identification of avian β-defensins in the zebra finch genome

All known amino acid sequences from AvBD of the mature peptide originating from the chicken genome [15,17,50] were used to search the Taeniopygia_guttata-3.2.4 (chromosomes) data base at http://genome.wustl.edu/tools/blast/ for homologous amino acid sequences using TBLASTN. All obtained amino acid sequences, regardless of E-value and degree of homology, were kept after the TBLASTN search. Each obtained hit was analysed by eye for the avian β-defensins characteristic cysteine motifs (X_n_-C-X_4-6_-C-X_3-5_-C-X_9-10_-C-X_5-6_-CC-X_n_, where X_x-x _represent the number of amino acids between the cysteines). All sequences containing this characteristic motif were saved and considered as AvBD candidates of the zebra finch genome. The corresponding nucleotide sequences were obtained from http://genome.ucsc.edu/cgi-bin/hgGateway based on the nucleotide positions on the chromosomes. All data were produced by the Genome Sequencing Centre at Washington University School of Medicine in St. Louis and can be obtained from: http://genome.wustl.edu/pub/organism/Other_Vertebrates/Taeniopygia_guttata/assembly/Taeniopygia_guttata-3.2.4/output/chromosomes/. For each of the found mature peptide sequences, the signal peptides (located in exon 2) could be identified upstream within a maximum of 2500 bp; most were found about 700 bp upstream. The signal peptides could easily be identified due to high similarity to the corresponding signal peptides in the chicken genome. All obtained gene locations and orientations were mapped and compared with the AvBD cluster in the chicken genome (figure 1). The different physical properties of the peptides were calculated using the on-line calculator at http://www.innovagen.se/custom-peptide-synthesis/peptide-property-calculator (table 1). Nucleotide sequence data reported are available in the Third Party Annotation Section of the DDBJ/EMBL/GenBank databases under the accession numbers TPA: BK006961-BK006982

### Expression analysis of β-defensin genes

454 pyro-sequencing reads from cDNA libraries constructed using six different zebra finch tissues (embryo, liver, muscle, skin, spleen and testes) were trimmed (for adaptor sequences, SMART primers, poly-A tails and low quality scores) and assembled using SeqMan NGgen 2.0 (DNASTAR, Inc). The cDNA libraries was pooled from approximately six healthy individuals without known pathogen exposure from the University of Sheffield population and contained cDNA from testis (340,347 sequences), day-9-embryo (366,151 sequences), muscle (356,890 sequences), spleen (329,135 sequences), liver (435,409 sequences), and skin (287,557 sequences) tissue [51]. A total of 1,882,439 reads with a mean read length of 83 base pairs (after trimming) were entered into the assembly. Out of these, 741,917 were assembled into 49,606 contigs while the rest were kept as singletons (for details about the assembly see Ekblom et al. submitted manuscript). All reads are available in the trace archive on the NCBI web site http://www.ncbi.nlm.nih.gov/Traces/home/. After having run a de-novo assembly using reads from all six tissues together, reads from each of the six tissues were assembled separately, using the contigs from the first assembly as template.

The nucleotide sequences of the signal peptide and the mature peptide, for each of the 22 annotated zebra finch β-defensin genes, were blasted (stand alone blastn version 2.2.18) against all contigs and singletons from the assembly of the 454 data. Four contigs and 274 singletons gave significant (E < 1e-05) hits (additional file 1). For each of these, only the best (lowest E-value and highest score) was extracted and run in a reciprocal blast against the zebra finch genome sequence. All putative β-defensin contigs and singletons gave highly significant (E < 1e-09) best blast hits against the zebra finch β-defensin gene cluster on chromosome 3 and the locus for each of the sequences was inferred from the chromosome coordinates of the best blast hit.

For each locus, the number of reads in contigs and singletons were counted for every tissue separately. Number of transcripts per million (TPM) and index of tissue specificity of gene expression (τ) [52] was calculated following the guidelines in [53]. Thus, the TPM was set to 2 for tissues without any detected expression for the gene in question and estimates of τ based on 3 or fewer reads in total were discarded. Theoretically τ varies from 0 to 1, with low values indicating even expression in all sampled tissues (house keeping genes), and high values for genes expressed solely in one of the tissues. Handling and analyses of blast results were performed in R version 2.7.2 [54].

### Phylogenetic reconstruction and evolutionary analysis

The full sequences were concatenated to obtain the signal peptide and the mature peptide. The corresponding amino acid sequences were then aligned using MUSCLE [55]. Nucleotide sequences were aligned correspondingly to the alignment of the amino acid sequences. Nucleotide sequences, together with chicken β-defensins (AvBD1-14) were used for phyolgenetic reconstruction in mrBayes 3.1.2. [56]. The run was partitioned to allow for the signal and the mature peptide to have different substitution rates and proportion of invariable sites. For both the signal peptide and the mature peptide, the run were allowed 6 different substitution rates and a gamma distribution with a proportion of invariable sites (corresponding to the GTR+I+Γ model). Two simultaneous runs were generated over 2000000 generations with a sampling frequency of every 100^th ^tree. Before analysis, 25% of the trees were discarded as burn-in period and the remaining trees were used to construct a majority consensus tree rooted on mid point. To get a second validation of the phylogenetic reconstruction, a neighbour-joining tree was constructed using MEGA 4.0 bootstrapped 10000 times under a Maximum Composite Likelihood Model [57]. Estimate of the branch length, using an all sites analysis could give duplicated genes proportional longer branches due to possible diversification events and difference in selection pressures on selective sites. To get a phylogeny with branch lengths that more represented the divergence time, we used the topology obtained from the baysian phylogeny and re-estimated the branch lengths using the dS matrix (i.e. the divergence between two lineages only using the synonymous sites) obtained from PAML [58](see below), using the fich module in PHYLIP 3.69 [59]. The obtained phylogeny was then imported into MEGA 4.0 [57] in order to compute a linearized tree that was rooted on mid point (figure 3).

First, a pairwise comparisons of the rate of synonymous substitutions (d_S_) and non-synonymous substitutions (d_N_) were calculated between all sequences using the method yn00 [60] as implemented in the software package PAML 4.0 [58] to get an initial estimate of d_S _and d_N_across the whole tree. Values obtained by ML as implemented in codonML in the software PAML 4 [58] was used when comparing the d_N_/d_S _(ω) ratios within phylogenetically robust clades (comparisons between recently duplicated genes and orthologous genes between the chicken and zebra finch). Two tests were conducted to investigate whether duplicated genes within the zebra finch genome have evolved under a different selection pressure compared to the homologous genes that not have been duplicated since the G-P split. First the ω values obtained by ML from the orthologous genes outside cluster A and B (figure 2) were compared with the ω value obtained between AvBD1 with the presumed related genes in cluster A and pairwise values between AvBD3 and its presumed related genes in cluster B. Secondly, to ensure that no errors was due to wrong phylogenetic relationships of AvBD1 and AvBD3 with the genes in cluster A and B, the same comparison was done with the exception that only duplicated genes grouped together with other intra-specific AvBD genes within a group of genes that were supported by a posterior probability higher than 90 was included (figure 2). The latter test gives an estimate of the mean ω after the duplication events, in contrast to the initial test that would also include a period during which the gene might have been evolving under stabilising selection before the duplication events. The comparisons of mean ω values were conducted using Mann-Whitney U tests and a Monte Carlo simulation based on 1000 sampled tables as implemented in SPSS 15.0.

CodeML in PAML [58] was used to investigate sites under positive selection in a dataset containing the genes found in cluster B in the zebra finch genome without the ortholog chicken gene in order to only include duplicated genes from one origin. If the whole duplicated cluster is inactive, or contains only one active gene, no positive selection should be observed. For this reason, the basal chicken gene was excluded, because otherwise the data set could potentially include two active genes but still having all duplicated genes inactive. Three different likelihood ratio tests (LRT) were conducted to investigate, which model of selection fitted the data best. Likelihood values was calculated for the following models (as recommended in the manual of PAML 4.0): M1a, a model that assume nearly neutral selection (i.e. ω = 1 or < 1), M2a (similar to M1a but with positive selection allowed, i.e. ω > or < 1), M7 (a more complex model of M1a with a β-distribution and ω can vary between 0-1), M8 (a more complex model of M2a with a β-distribution and ω that can obtain values > or < than 1), M8a (similar to M8 but ω_s _= 1). The combination of LRTs where M1a vs. M2a, M7 vs.M8 and M8a vs. M8. The models M2 and M8 calculate any sites under positive selection with a 95% and 99% posterior probability using Bayes empirical Bayes (BEB).

## Competing interests

The authors declare that they have no competing interests.

## Authors' contributions

OH carried out the molecular genetic studies, phylogenetic analysis, performed the statistical analysis and drafted the manuscript. RE carried out the gene expression analysis and helped to draft the manuscript. All authors read and approved the final manuscript.

## Supplementary Material

Additional file 1 **Contigs and singleton reads of defensin genes obtained from zebra finch**. Supplemental FASTA file. Click here for file
